# Synthesis and Characterization of Maleic Anhydride-Methyl Methacrylate Co-Monomer Grafted Polyethylene Wax for Hot Waxed Wood Process

**DOI:** 10.3390/ma15196962

**Published:** 2022-10-07

**Authors:** Kangren Niu, Kuiyan Song

**Affiliations:** Key Laboratory of Bio-Based Material Science and Technology of the Ministry of Education, School of Materials Science and Engineering, Northeast Forestry University, Harbin 150040, China

**Keywords:** beeswax, polyethylene wax, grafting, maleic anhydride, methyl methacrylate, thermal and chemical properties

## Abstract

The beeswax used in Chinese traditional hot waxed wood technology has several drawbacks, such as high price, scarce resources, and poor heat resistance. To expand the application of hot waxing technology in the field of wood decoration and protection, polyethylene wax was modified by a grafted maleic anhydride-methyl methacrylate co-monomer. A type of modified polyethylene wax with low cost, high melting point, high stability, and strong polarity suitable for hot waxed wood was prepared as a replacement for beeswax. The effects of the grafting conditions on the chemical properties, thermal properties, chemical structure, and crystallization properties of modified polyethylene wax were studied and compared to those of beeswax. The results show that maleic anhydride-methyl methacrylate co-monomer grafting can effectively improve the acid value of polyethylene wax. For the ratio of two monomers of 1/1, the total amount of monomer of 8 wt%, the amount of initiator of 2 wt%, the reaction temperature of 150 °C, and the acid value of modified polyethylene wax was consistent with that of beeswax, realizing the simulation of the main chemical properties of beeswax. The thermal stability and melting temperature of the modified polyethylene wax are significantly higher than those of beeswax, its crystal structure is similar to that of beeswax, and the cyclic anhydride groups and ester groups introduced by co-monomer grafting endow it with polar groups that play an important role in the wood hot waxing process.

## 1. Introduction

Wood decoration by hot waxing with beeswax has long been used in China and is a simple and environmentally friendly method for the surface waterproofing treatment of hardwood furniture. Although the beeswax used in the traditional hot waxed wood process has the advantages of good gloss and good compatibility, its wide use in modern industrial production is hindered by its high cost and shortage of resources [1]. In addition, the low melting point of beeswax leads to the problem of the poor heat resistance of the beeswax hot waxed wood. The replacement of beeswax with synthetic waxes, e.g., polyolefins with a wide range of sources, low cost, and high melting points can provide a new approach for expanding the application of the hot waxing technology in the field of wood decoration and protection. In recent years, increasing research attention has been devoted to the application of synthetic waxes in wood protection where the wood is usually impregnated with a wax emulsion to improve its hydrophobicity, dimensional stability, and mechanical strength [2,3,4,5,6,7]. However, polar groups are almost entirely absent in synthetic waxes, hindering the interactions of the waxes with the polar groups of wood fiber. This leads to several problems for the use of synthetic wax instead of beeswax in the hot waxed wood process, such as low adhesion after wax coating film formation and poor flexibility. Therefore, the introduction of polar groups into the main chain of synthetic wax by grafting modification is beneficial for improving its compatibility with other polar polymers and addressing the lack of toughness of synthetic wax.

Grafting is considered to be an important technology for modifying the physical and chemical properties of polymers. Free radical or anionic grafting methods are used in the grafting modification of polymers [8,9]. Free-radical grafting is the grafting of small molecular functional groups onto polyolefin molecular chains. Various monomers, such as maleic anhydride (MAH) [10,11], acrylic acid (AA) [12], amide [13], N-vinyl pyrrolidone [14], aminoethyl methacrylate (AEMA) [15], chitosan [16], and polyethylene glycol (PEG) [17], have been used for the grafting modification of polyolefins. Among these, MAH-grafted polyolefin in the presence of organic peroxides has attracted intense attention. Maleic anhydride derives its name from naturally occurring malic acid. Maleic anhydride is an important organic chemical raw material used in the polyester resin, alkyd resin, surface coating, lubricating oil additive, plasticizer (QV), fumaric acid, and so on [18]. Because MAH does not easily homopolymerize during free-radical grafting and the unsaturated double bonds contained in MAH can react with hydroxyl (-OH), carboxyl (-COOH), and amino (-NH_2_) groups, the compatibility of polyolefin with other polar polymers is enhanced. MAH has been successfully grafted onto polyolefin molecular chain by solution [19,20,21,22,23], melts [22,24,25,26,27,28,29], and solid-state routes [22,27,30,31]. In addition, grafting reactions induced by ultraviolet (UV) irradiation, high-energy radiation (such as γ-ray), and photografting have also been reported [22].

However, the grafting degree of MAH on polyolefins is usually quite low. This is due to the low reactivity of MAH to free radicals and its low solubility in the polyolefin matrix. The low free-radical reactivity of MAH is due to its intrinsic structural symmetry and electron density deficiency around the double bond [32]. To obtain a high degree of grafting, it is essential for the reaction of the MAH monomers with polyolefin macroradicals to occur prior to undergoing side reactions (e.g., cross-linking and chain scission) [33]. The reactive end group of MAH can be functionalized with heterogeneous monomers, providing favorable conditions for multi-monomer grafting to improve the activity of MAH. Co-monomers usually must have higher activity toward both of the polyolefin macroradicals and the MAH monomer. In the study of single and oligomeric grafting of MAH onto polyolefin, Gaylord et al. [34,35] reported that styrene can be alternately copolymerized with MAH by charge transfer complexes. Therefore, the gap between macroradicals and MAH monomers can be bridged by styrene, thus increasing the degree of grafting of MAH on polyolefins.

In this study, methyl methacrylate (MMA)-assisted grafting MAH was selected to modify polyethylene wax. Because of the low reactivity ratio of MMA and MAH [12], it easily forms alternating copolymerization with MAH, and it is possible to obtain a polyethylene wax with polar groups that is similar to beeswax. The acid value, thermal properties, chemical structure, and crystallization properties of the modified polyethylene wax were analyzed by titration, thermogravimetric analysis (TGA), differential scanning calorimetry (DSC), Fourier transform infrared spectroscopy (FTIR), proton nuclear magnetic resonance spectroscopy (^1^H-NMR), and X-ray diffraction (XRD). The effects of the ratio of grafting monomers, the number of monomers, the amount of initiator, and reaction temperature on the properties of the product and the grafting mechanism were investigated. Based on these investigation and comparison with the related properties of beeswax, the modified polyethylene wax with high melting point, high stability, and strong polarity suitable for hot waxed wood process was prepared as a replacement for beeswax.

## 2. Experimental

### 2.1. Materials

Beeswax (BW) was taken from the Dengfeng Bee Farm in Henan Province, China. Samples were taken from the center of the wax block and heated and melted in distilled water to filter impurities. Polyethylene wax (PEW), produced by Thailand SCG, Co. (Bangkok, Thailand), was dissolved and refluxed with xylene at 120 °C for 2 h, and then methanol was added to precipitate and purify it. Analytical grade maleic anhydride (MAH) was purchased from Tianjin Guangfu Fine Chemical Research Institute (Tianjin, China). Analytical grade methyl methacrylate (MMA) was purchased from Aladdin Reagent Co., Ltd. (Shanghai, China). Analytical grade benzoyl peroxide (BPO) with a half-life time of 1 min at 131 °C was purchased from Shanghai McLean Biochemical Technology Co., Ltd. (Shanghai, China). Analytical grade xylene and methanol were provided by Tianjin Fuyu Fine Chemical Co., Ltd. (Tianjin, China). The above reagents were used without further purification. 

### 2.2. Preparation of Polyethylene Wax Modified by Grafted Maleic Anhydride-Methyl Acrylate (PEW-g-MAH-MMA)

A certain mass of purified polyethylene wax and xylene with twice the mass of polyethylene wax were poured into a four-port bottle and vibrated several times for full mixing of polyethylene wax and xylene. The four-port bottle was poured into a constant temperature oil bath and connected to a reflux condensing tube, a thermometer, a slurry electric agitator, and gas conduit. Then, a nitrogen purge with a nitrogen flow rate of 20 mL min^−1^ was carried out to obtain nitrogen atmosphere for the four-port bottle. 

The oil bath was heated to 120 °C and stirred at a constant speed of 170 rpm to ensure that the polyethylene wax was completely melted and mixed with xylene. Then, the pre-prepared BPO initiator and MAH/MMA xylene solution were added dropwise to the polyethylene wax/xylene mixture stirred at constant speed (360 rpm) using a constant-pressure separation funnel, and the mixture reacted for 3 h at 130–160 °C. The details of the procedure and grafting temperatures are shown in Table 1. 

**Figure 1:**
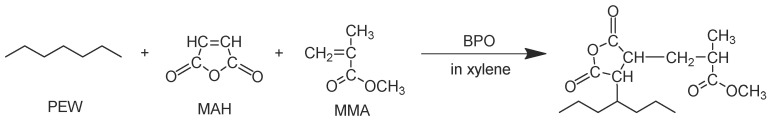
The reaction equation of polyethylene wax (PEW) grafted with maleic anhydride (MAH) and methyl methacrylate (MMA).

At the end of the reaction, the reaction solution was agitated rapidly, and the crude product was precipitated with methanol. The mass of methanol was three times higher than the mass of polyethylene wax. After methanol washing and filtration, the crude product was soaked in xylene for 24 h to extract the unreacted MAH/MMA monomers, MAH/MMA homopolymers, and other impurities. Purified graft maleic anhydride-methyl methacrylate modified polyethylene wax (PEW-*g*-MAH-MMA) was obtained by vacuum drying at 50 °C for 12 h. The reaction equation for the grafting of MAH-MMA onto PEW is shown in Figure 1.

### 2.3. Acid Value Tests

The acid values of BW, PEW, and PEW-*g*-MAH-MMA with different grafting conditions were determined by titration. According to the ASTM D1386-15 standard, a wax sample (1 g, accurate to 0.001 g) was placed into a 250 mL flask, xylene solution (40 mL) was added, and the sample was refluxed and dissolved in an oil bath until the sample was clear and transparent. Three to five drops of phenolphthalein solution were added and the solution was titrated to pink with a standard KOH solution (0.05 mol/L) with the color not fading within 10 s indicating the titration end point. The flask was shaken violently during titration and the sample was reheated if precipitation occurred during the titration. The number of milliliters of KOH standard solution used for titration was recorded and a blank test was performed at the same time. The acid value (KOH mg/g) was calculated by Equation (1). The experiments were repeated five times for each group of wax samples, and the average value was taken as the final result.

Acid number (%) = (*A − B*) × *N* × 56.1/*C* (1)

where *A* is the milliliters of the KOH standard solution required for titration of the sample, *B* is the milliliters of KOH standard solution required for titration of the blank sample, *N* is the normality of KOH standard solution, and *C* is the grams of the tested wax sample. 

### 2.4. Mechanical Properties Tests

According to the ASTM D882-02 standard, the tensile strength and elongation at break of PEW and PEW-*g*-MAH-MMA were determined using a Universal Test Machine (LABSANS, Shenzhen, China). The samples were cut into strips with dimensions of 1 × 8 cm and conditioned at 23 °C and 50%RH for at least 24 h. The samples were tested at a crosshead speed of 10 mm·min^−1^. Each sample was repeated 5 times, and the average value was taken as the final result.

### 2.5. Thermal Stability Tests

The thermogravimetric analysis of BW, PEW, and PEW-*g*-MAH-MMA was carried out using a thermogravimetric analyzer (TGA, STA449 F3, Netzsch, Germany) to characterize the thermal stability of different wax samples. The sample (5–10 mg) was weighed each time and heated from 30 °C to 600 °C at a heating rate of 10 °C min^−1^ under nitrogen atmosphere (50 mL min^−1^). The derivative mass loss (DTG) curves were obtained, and the thermal stability of the wax sample was evaluated by the temperature corresponding to the maximum thermal weight loss from the DTG curve. 

### 2.6. Differential Scanning Calorimetry (DSC) Tests

The thermal properties of the BW, PEW, and PEW-*g*-MAH-MMA samples were studied using a DSC-60 differential scanning calorimeter (Shimadzu, Kyoto, Japan). The sample weight was 5–10 mg, and the experiments were performed in nitrogen atmosphere. The test procedure is divided into three steps: (1) heating from 25 °C at a heating rate of 10 °C min^−1^ to 160 °C and equilibrating for 5 min to eliminate the thermal history; (2) naturally cooling the temperature from 160 °C to 25 °C; and (3) reheating to 160 °C at 10 °C min^−1^. The reheating DSC curves were recorded to study the thermal behavior of the samples. 

### 2.7. Fourier Transform Infrared Spectroscopy (FTIR) Tests 

Attenuated total reflection Fourier transform infrared spectroscopy (ATR-FTIR) was performed on a Nicolet is50 FTIR spectrometer (Thermo Fisher Scientific, Waltham, MA, USA). The active functional groups of BW, PEW, and PEW-*g*-MAH-MMA samples were analyzed by Fourier transform infrared spectroscopy. The spectra were collected with an average of 32 scans at a resolution of 4 cm^−1^ in the wavenumber range of 400–4000 cm^−1^ in the transmittance mode. 

### 2.8. Proton Nuclear Magnetic Resonance Spectroscopy (^1^H-NMR) Tests

PEW and PEW-g-MAH-MMA samples were prepared in tetrachloroethane solution and sealed in NMR tubes. Proton nuclear magnetic resonance (^1^H-NMR) spectroscopy was recorded at 600 MHz operating frequency using ^1^H NMR spectrometer (Bruker Avance NEO 600, Billerica, MA, USA) at 120 °C.

### 2.9. X-ray Diffraction (XRD) Tests

The crystallization properties of the BW, PEW, and PEW-*g*-MAH-MMA samples were analyzed by X-ray diffractometry (XRD, Empyrean, Panalytical, Almelo, The Netherlands) using Cu target Kα radiation, an X-ray wavelength of 0.154 nm, a radiation tube voltage of 40 kV, a tube current of 40 mA, a scanning angle range of 10–50°, a scanning speed of 4°/min, and a step of 0.02°. The degree of crystallinity of sample was calculated by dividing the total area under Bragg peaks on the total area under Bragg peaks plus the area under hump.

## 3. Results and Discussion

### 3.1. Chemical Structure Analysis

**Figure 2:**
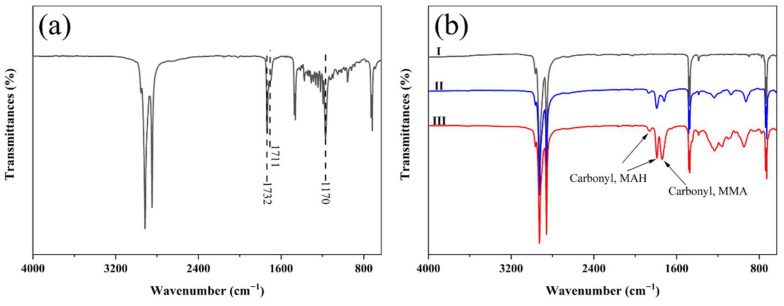
FTIR spectra of (**a**) BW, (**b**) PEW (I), PEW-*g*-MAH (II), and PEW-*g*-MAH-MMA (III).

Figure 2a shows the FTIR spectrum of BW. BW is a complex organic mixture that is composed of many compounds. The main characteristic bands observed in the FTIR spectra of BW are related to the hydrocarbons, esters, and free fatty acids in its structure. The strong peaks at 2916 cm^−1^ (shoulder peak at 2955 cm^−1^) and 2848 cm^−1^ are due to the asymmetric and symmetric C-H stretching of aliphatic methylene (-CH_2_-) and methyl (-CH_3_). The absorption peaks near 1473 cm^−1^ and 1463 cm^−1^ are due to the bending vibrations of -CH_3_ and -CH_2_-. The absorption peaks near 730 cm^−1^ and 719 cm^−1^ are attributed to the in-plane wagging vibrations of -CH_3_ and -CH_2_-. The peak at 1732 cm^−1^ is due to the stretching vibration of the unconjugated carbonyl groups in esters, the peak at 1711 cm^−1^ arises from the vibrations of unconjugated carbonyl groups in free fatty acids, and the peak at 1170 cm^−1^ is due to the C-O vibration in esters. Similar FTIR spectra of BW have been reported in the literature [1,36,37,38,39,40,41].

Compared to the FTIR spectrum of BW, the FTIR spectrum of PEW (Figure 2b, Spectrum I) reflects the simple molecular structure related to the absorption band of hydrocarbons (at 2955, 2916, 2849, 1473, 1462, 730, and 719 cm^−1^). Clear differences between the spectra of PEW and BW are observed in the fingerprint region. There is no absorption band related to esters and free fatty acids in PEW. Different from the FTIR spectrum of PEW, in the FTIR spectrum of PEW-*g*-MAH (Figure 2b, Spectrum II), three additional absorption peaks at 1861, 1782, and 1711 cm^−1^ are observed. The peaks at 1861 cm^−1^ and 1782 cm^−1^ are attributed to the asymmetric and symmetric stretching vibrations of cyclic anhydride carbonyl (C=O) [42,43,44], and the peak at 1711 cm^−1^ is attributed to the stretching vibration of the carbonyl groups in the carboxylic groups [42]. These results showed that MAH was grafted onto PEW. Compared to the FTIR spectrum of PEW-*g*-MAH, the FTIR spectrum of PEW-*g*-MAH-MMA (Figure 2b, Spectrum III) shows the peaks of the carbonyl groups in the five-membered anhydride ring at 1849 cm^−1^ and 1781 cm^−1^, and an additional characteristic peak at 1731 cm^−1^ that can be attributed to the stretching vibration of the carbonyl groups on MMA [45]. It was observed that there was no absorption peak at 1637 cm^−1^ due to C=C vibrations, indicating that MAH and MMA were copolymerized and grafted onto the macromolecular chain of PEW. In addition, the characteristic peak strength of the anhydride groups of PEW-*g*-MAH-MMA was significantly higher than that of PEW-*g*-MAH. These results further confirmed the effectiveness of co-monomer grafting in improving the grafting degree of MAH. 

**Figure 3:**
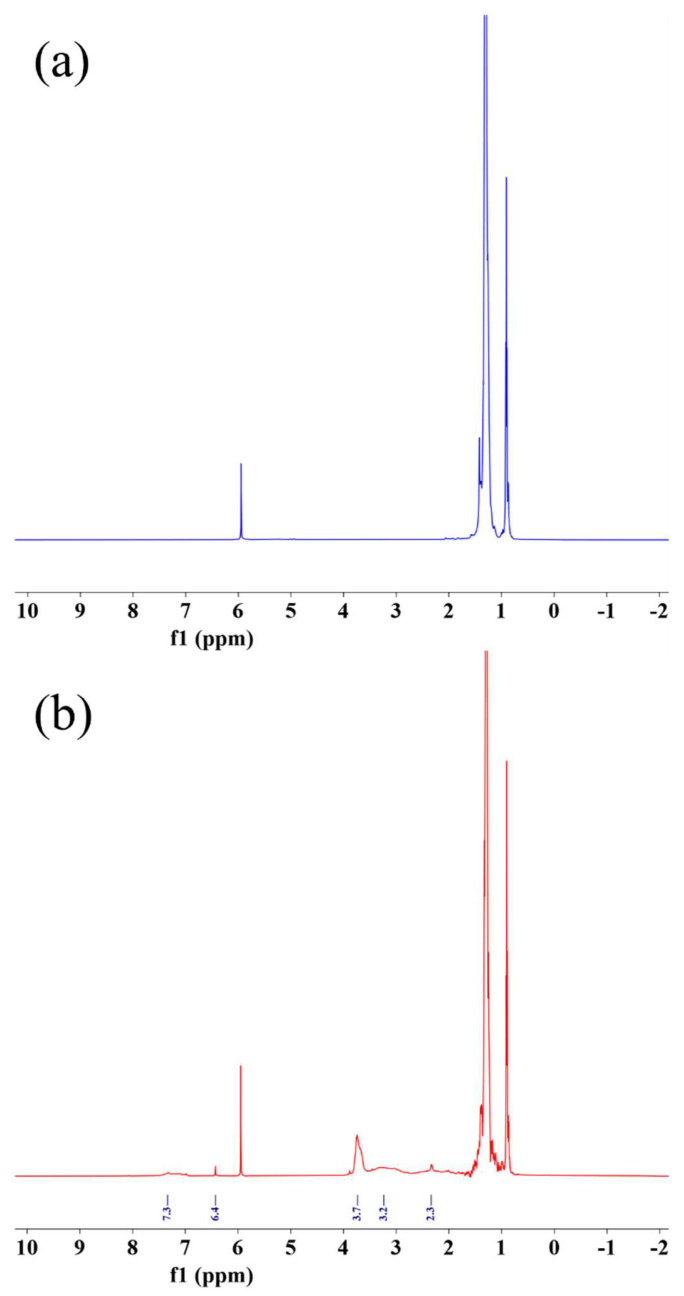
^1^H NMR spectra of PEW sample (**a**) and PEW-*g*-MAH-MMA sample (**b**).

Figure 3 compares the ^1^H-NMR spectra of PEW-*g*-MAH-MMA with the corresponding PEW, and further analyzes the structure of MAH-MMA grafted PEW by ^1^H-NMR. Compared with PEW, PEW-*g*-MAH-MMA has two new chemical shifts at 3.7 and 3.2 ppm. 3.2 is the proton peak of methylene (-CHCH) in MAH structural unit, and 3.7 belongs to the proton peak of methyl hydrogen on -COOCH_3_ in the MMA structural unit [46,47], indicating that MAH and MMA were copolymerized and grafted onto PEW. In addition, compared with the proton peak of hydrogen on the unsaturated bond in raw MAH, which is 6.4, the proton peaks of hydrogen on the unsaturated bond are 6.00 and 5.4 ppm in raw MMA, it is considered that the basic reaction of MAH and MMA double bonds in the product is complete, only a weak peak is found at 6.4 ppm, indicating the existence of trace amounts of MAH. The weak peaks at 2.3 and 7.3 ppm in the ^1^H-NMR spectrum belong to the proton peaks in the xylene solvent and have not been completely removed from the product. ^1^H-NMR analysis further proved that MAH and MMA formed a copolymer and grafted onto PEW.

### 3.2. Acid Value Analysis

**Figure 4:**
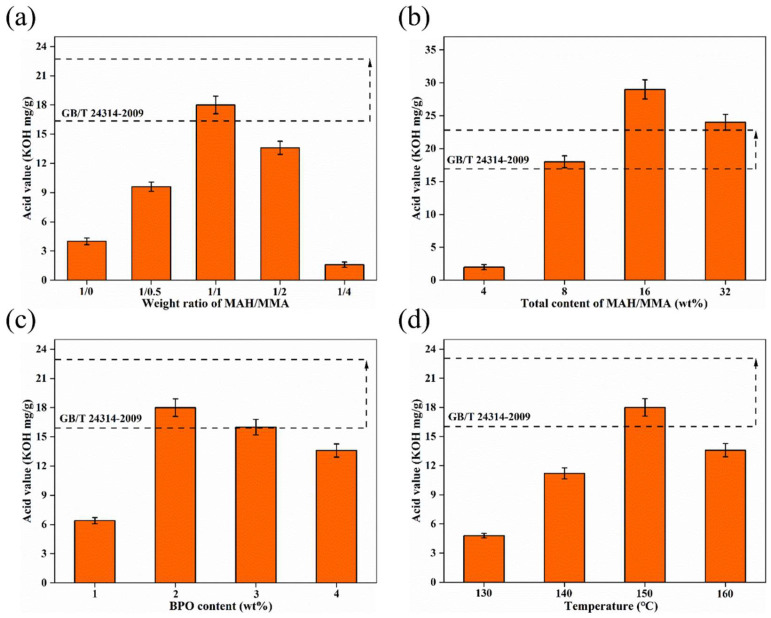
Effect of (**a**) weight ratio of MAH/MMA, (**b**) total content of MAH/MMA, (**c**) content of BPO, and (**d**) reaction temperature on the acid value of modified polyethylene wax.

According to the physical and chemical requirements for BW products in the GB/T 24314-2009 national standard, the acid value is the most important chemical property of BW that directly reflects the polarity and advantages and disadvantages of BW products. To make the chemical properties of PEW-*g*-MAH-MMA similar to those of BW, acid value was selected as the key indicator of the performance of PEW-*g*-MAH-MMA. Due to the hydrolysis of maleic anhydride (MAH) to maleic acid, PEW-*g*-MAH-MMA also showed acidity. Based on a large number of previous experiments, the acid value of PEW-*g*-MAH-MMA is affected by the formulation parameters and process conditions. This section studies the relationship between the acid value of PEW-*g*-MAH-MMA and the weight ratio of MAH/MMA, the total amount of monomer, the amount of initiator (BPO), and reaction temperature. The results are shown in Figure 4.

The effect of the weight ratio of MAH and MMA on the acid value of PEW-*g*-MAH-MMA is shown in Figure 4a. In the absence of MMA, the acid value is low. With the increase in the MMA content, the acid value of PEW-*g*-MAH-MMA increases gradually. The maximum acid value is obtained for the MAH/MMA weight ratio of 1/1. When the MMA content was higher than that of MAH, the acid value decreased gradually, and when the weight ratio of MAH/MMA was 1/4, the acid value decreased sharply, and was even lower than that of the MAH grafted alone. Because of its symmetrical structure and the low electron density around the -CH=CH- bond [33,48], MAH has low reaction activity to PEW macroradicals, and it is difficult to form a graft chain on the macromolecular chain of PEW, resulting in its low acid value. However, the reactive end group of MAH can be functionalized with heterogeneous monomers that provide favorable conditions for multi-monomer co-grafting. When MMA is added, because the reactivity ratio of MMA and MAH is small [12], the interaction of MMA and MAH can easily form alternating copolymers. The formation of alternating copolymers enhances the electrical asymmetry of the -CH=CH- bond in MAH improves the reaction activity of the monomers and makes it easy to combine with PEW macroradicals, thus forming an MAH-MMA alternating copolymer graft chain on the PEW macromolecular chain. Therefore, the acid value of PEW-*g*-MAH-MMA was significantly increased by the addition of MMA. It is observed from the Figure 4a that the MAH/MMA weight ratio has a significant effect on the acid value of PEW-*g*-MAH-MMA, and the mechanism of MMA-assisted MAH grafting PEW depends on the MAH/MMA ratio.

(1) Because of the similarity of the molar mass of MAH and MMA, for the addition of MAH and MMA with the same molar amount (the molar mass of MAH/MMA is similar, the weight ratio is similar to the molar ratio), the two monomers can easily interact with each other. Theoretically, all MAH and MMA can form MAH-MMA copolymers, thus forming a longer branching structure on the PEW macromolecular chain and resulting in an increase in the number of grafted anhydrides. Therefore, the highest acid value of PEW-*g*-MAH-MMA is reached when the MAH/MMA ratio reaches 1/1. (2) For the MAH/MMA ratio > 1/1, a fraction of MAH forms a copolymer with MMA. The MAH/MMA copolymer is seldom generated because of the low MMA content. Therefore, the grafting form of the PEW macromolecular chain is mainly a single MAH and MAH-MMA copolymer graft. Therefore, for the MAH/MMA ratio of 1/0.5, the acid value of PEW-*g*-MAH-MMA is between those of MAH/MMA (1/0) and MAH/MMA (1/1). (3) For the MAH/MMA ratio less than 1/1, some MMA monomers react with MAH to form MAH-MMA copolymers, while excess MMA monomers will preferentially react with PEW macroradicals, consuming primary radicals and PEW macroradicals and resulting in a decrease in the amount of grafted anhydride and the acid value. In addition, with the further increase in the MMA content, due to the easy self-polymerization of MMA [42,49], the self-polymerization of MMA is dominant at this stage. Therefore, for the MAH/MMA ratio of 1/4, the acid value of PEW-*g*-MAH-MMA is lower than that of the MAH grafted alone. 

A MAH/MMA ratio of 1/1 was applied for other formulations because it gave the highest acid value. Figure 4b shows the effect of the total content of the MAH/MMA monomer (as a percentage of the weight of PEW) on the acid value of PEW-*g*-MAH-MMA. With the increase in the amount of the monomer, the acid value increases. When the total amount of monomer is in the range of 4–8 wt%, the acid value increases greatly. When the amount of monomer is greater than 8 wt%, a smaller increase in the acid value is obtained. However, with the further increase in the amount of monomer, when the amount of monomer reached 32 wt%, the acid value of PEW-*g*-MAH-MMA clearly decreased.

The process of radical grafting actually starts with the decomposition of BPO to form primary radicals that then initiate PEW macroradicals by hydrogen abstraction [10]. Then, macroradicals participate in the grafting reaction to initiate the grafting of MAH-MMA co-monomers, but PEW macroradicals are accompanied by side reactions, such as cross-linking [50]. When the content of BPO is fixed, the number of grafting sites produced by the PEW macromolecular chain is relatively fixed, and when the amount of the monomer is small, the probability of a collision between the graft monomer and PEW macroradicals is small. By contrast, the probability of side reaction of the macroradicals increases, which is not conducive to the grafting reaction. Therefore, the acid value of PEW-*g*-MAH-MMA is low. The increase in the amount of the monomer helps to increase the likelihood of the collision with macroradicals and fully exploits the radical initiation of monomer grafting. Therefore, the acid value of PEW-*g*-MAH-MMA increases with increasing monomer content. However, a too high monomer content may give rise to the shielding effect and reduce the initiation efficiency of the radicals. In addition, a too high monomers content will increase the probability of side reactions, such as homopolymerization; reduce the amount of actual grafted anhydride; and lead to a decrease in the acid value. 

The effect of the content of BPO on the acid value of PEW-*g*-MAH-MMA is shown in Figure 4c. It can be observed from the figure that the acid value of PEW-*g*-MAH-MMA increases at first and then decreases slowly with the increase of the amount of BPO. When the amount of BPO reaches 2 wt%, the acid value of PEW-*g*-MAH-MMA is the highest. For a small amount of BPO, the number of radicals in the grafting reaction system is insufficient to initiate the grafting reaction, resulting in a small number of grafted anhydride and a low acid value. With the increase in the amount of BPO, the number of primary radicals increased, so that more PEW macroradicals were produced by the hydrogen extraction reaction, which is beneficial for the full initiation of the graft polymerization of MAH-MMA monomers. 

Therefore, increasing the amount of BPO in a certain range is an effective approach for increasing the acid value of PEW-*g*-MAH-MMA. However, the amount of BPO is not only directly related to the number of radicals but also related to the degree of cross-linking of the PEW molecules [43,51]. With the further increase in the amount of BPO, excessive BPO increased the probability of radical termination, giving rise to an increased degree of cross-linking of PEW molecules. In addition, a too large amount of BPO increases the probability of the homopolymerization of the monomers, hindering the grafting reaction and reducing the acid value of PEW-*g*-MAH-MMA.

Figure 4d shows the effect of the reaction temperature on the acid value of PEW-*g*-MAH-MMA. The acid value of PEW-*g*-MAH-MMA increases with increasing reaction temperature, and reaches the maximum at 150 °C. However, after the acid value reaches the maximum, the further increase of reaction temperature does not increase the acid value of PEW-*g*-MAH-MMA. On the contrary, the acid value decreased clearly at 160 °C. The process of thermal decomposition of BPO for the production of primary radicals is the initial and the most critical stage of the grafting reaction. The reaction temperature is closely related to the decomposition rate (activation energy and half-life) of BPO [32,41,51]. At lower reaction temperatures, the decomposition of BPO is slower, its half-life is long, and fewer reaction active centers are produced in the reaction process, resulting in a small number of macroradicals. In addition, low temperatures lead to a high solution viscosity that is unfavorable for the movement of radicals, thus reducing the collision probability of the macroradicals with grafted monomers and resulting in a small number of grafted anhydrides and low acid value. 

With increasing temperature, the rate of BPO decomposition increases, its half-life shortens, and the number of the reaction active centers increases, thus leading to the formation of more macroradicals. In addition, an increase in the temperature is beneficial for the movement of radicals and increases the collision probability of the macroradicals with grafted monomers and leads to the increase in the acid value. However, when the temperature is too high, the BPO decomposition rate is too high and its half-life is too short, resulting in a sharp increase in the number of macroradicals in a short time, thus aggravating the cross-linking of PEW molecules. Due to the influence of steric hindrance, the activation energy of the cross-linking reaction is higher than that of the grafting reaction, making the cross-linking reaction more sensitive to temperature. At higher temperatures, the aggravation of the cross-linking reaction will consume some macroradicals, which is unfavorable for the grafting reaction. In addition, a too high temperature may also lead to the increase in the chain transfer constant of the solvent and the decrease of the initiation efficiency of BPO, leading to the decreased acid value of PEW-*g*-MAH-MMA.

According to the acid value test results of BW and PEW-*g*-MAH-MMA with different grafting conditions shown in Table 2, for the MAH/MMA ratio of 1/1, the total amount of monomer of 8 wt%, the BPO concentration of 2 wt%, and the reaction temperature of 150 °C, the acid value of PEW-*g*-MAH-MMA reaches 18 KOH mg/g, which is consistent with BW and conforms to the national standard. The following characterization and testing of PEW-*g*-MAH-MMA samples, unless otherwise specified, were performed on the samples prepared with these parameters and process conditions. 

### 3.3. Mechanical Properties Analysis

**Figure 5:**
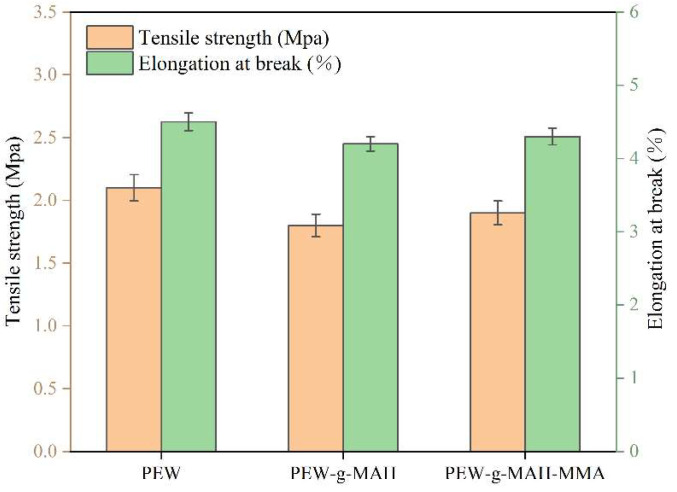
Tensile strength and elongation at break of PEW, PEW-*g*-MAH, and PEW-*g*-MAH-MMA.

Figure 5 shows the tensile strength and elongation at break of PEW, PEW-*g*-MAH, and PEW-*g*-MAH-MMA. Compared with PEW, the tensile strength and elongation at break of PEW-*g*-MAH and PEW-*g*-MAH-MMA decreases only slightly, and the difference of mechanical properties between samples is not significant, indicating that the introduction of MAH and MAH-MMA has no obvious effect on the mechanical properties of polyethylene wax.

### 3.4. Thermogravimetric Analysis

**Figure 6:**
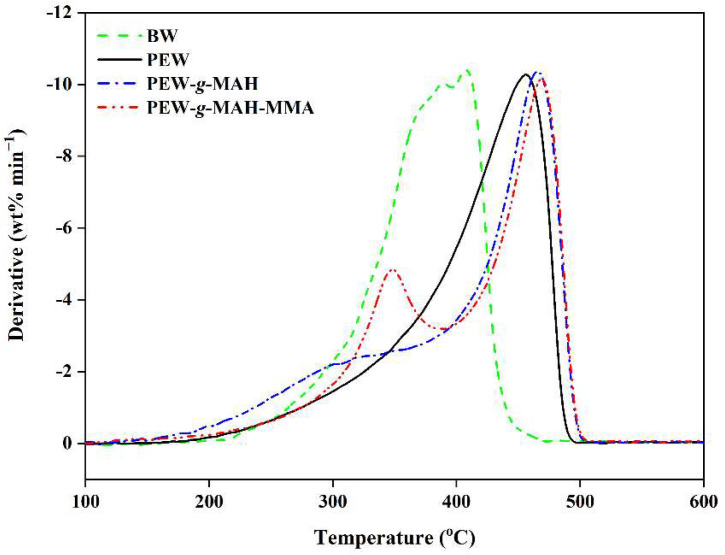
The derivative mass loss (DTG) curves of BW, PEW, PEW-*g*-MAH, and PEW-*g*-MAH-MMA.

Figure 6 shows the thermal decomposition results for BW, PEW, PEW-*g*-MAH, and PEW-*g*-MAH-MMA as a function of temperature. It is observed that the maximum thermal weight loss temperatures of BW and PEW are 400 °C and 450 °C, respectively. The results for the thermal decomposition and volatilization of BW and PEW indicate that the thermal stability of PEW is higher than that of BW. Compared to PEW, the maximum thermal weight loss temperature of PEW-*g*-MAH shifted to the high temperature and reached the maximum thermal weight loss at 455 °C. In addition, the DTG curve of PEW-*g*-MAH also showed a broad peak at 316 °C, which may be attributed to the thermal decomposition of the PEW-grafted MAH monomer.

It was found that MAH grafting improves the thermal stability of PEW. This may be attributed to the following effects. First, the introduction of the MAH monomer into the macromolecular chain of PEW increases the molecular weight of PEW and generates a certain number of branched chains on the macromolecular chain of PEW that hinder the thermal movement of the PEW molecular chain to some extent. Second, the five-membered ring structure of the grafted MAH monomer increases the rigidity of the PEW molecular chain, interfering with thermal degradation and improving the heat resistance of PEW. Compared to the DTG curve of PEW-*g*-MAH, the temperature corresponding to the maximum thermal weight loss of PEW-*g*-MAH-MMA increased and reached the maximum thermal weight loss at 462 °C. In addition, the peak at approximately 348 °C is attributed to the thermal decomposition of the MAH-MMA co-monomer grafted with PEW. It is clear that the effect of the MAH-MMA co-monomer grafting on the thermal stability of PEW is higher than that of the MAH grafting alone, which may be due to the increase in the grafting amount of MAH by the addition of MMA. In addition, the increase in the number of anhydride groups is beneficial for enhancing the interaction between the anhydride groups and may hinder the decomposition and volatilization of the monomers to some extent. 

### 3.5. Melting Behavior Analysis

**Figure 7:**
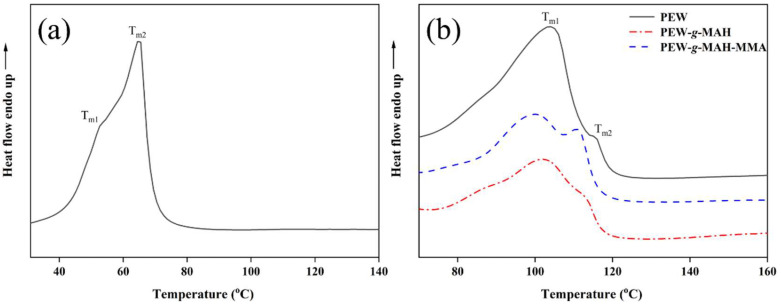
DSC secondary heating curves of (**a**) BW (**b**) PEW, PEW-*g*-MAH, and PEW-*g*-MAH-MMA. In the figure, T_m1_ and T_m2_ are the melting peak temperatures of the wax samples.

Figure 7a shows the DSC curve of the heat flow as a function of temperature for BW. The DSC heating curve is taken from the second heating scan to eliminate thermal history. It is observed that BW shows a main peak at 62 °C and a shoulder peak at lower temperature. These results are consistent with the results reported in the literature [36,52]. The main peak is caused by the solid–liquid transition, and the shoulder peak can be attributed to the solid–solid transition.

Similar to the DSC curve of BW, double melting peaks were also observed in the DSC curve of PEW (Figure 7b). However, the melting peak temperatures (T_m1_ and T_m2_) of PEW were significantly higher than those of BW and were found to be 104 °C and 115 °C, respectively. The first peak is related to the solid phase transition of the structure of PEW, and the second peak is related to the melting transition [53,54]. The results show that the molecular chain of PEW has higher mobility and higher melting transition temperature. The DSC curves (Figure 7b) of PEW-*g*-MAH and PEW-*g*-MAH-MMA are similar to that of PEW, but the melting peak temperature is slightly lower than that of PEW, and T_m1_ and T_m2_ decrease by 2–4 °C. The difference in the melting peak temperature between PEW-*g*-MAH and PEW-*g*-MAH-MMA is not significant, indicating that the existence of MMA has no obvious effect on the melting behavior of MAH-grafted PEW. The decrease in the melting peak temperature may be due to the following reasons. First, the grafting of the anhydride groups on the PEW macromolecular chain increases the amorphous region in the PEW. Motaung et al., have obtained similar findings in the study of MAH grafted low density polyethylene [55]. Second, the steric hindrance of the grafted side chain restricts the crystallization of PEW to a certain extent. The increase of the amorphous fraction and the decrease of crystalline fraction leads to a small decrease in the melting temperature of PEW.

### 3.6. Crystallization Property Analysis

**Figure 8:**
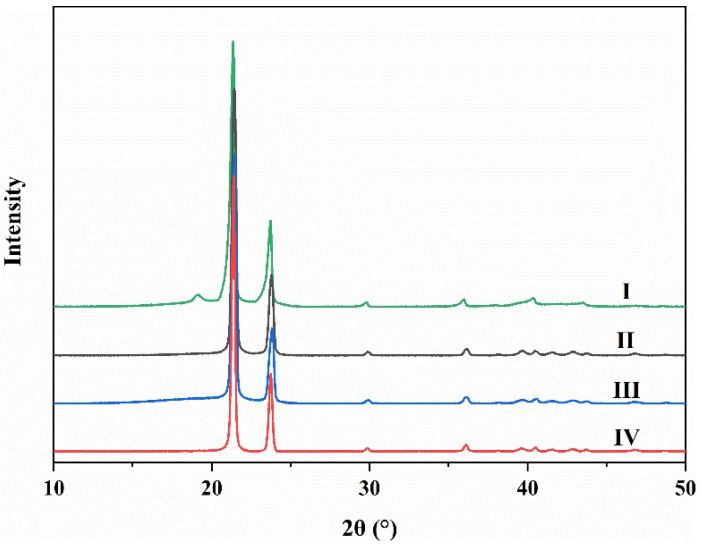
X-ray diffraction spectra of BW (I), PEW (II), PEW-*g*-MAH (III), and PEW-*g*-MAH-MMA (IV).

Figure 8 shows the XRD patterns of BW, PEW, PEW-*g*-MAH, and PEW-*g*-MAH-MMA. From the XRD curve of BW (Figure 8, Curve I), it is observed that BW displays two strong and clear separation peaks at 2θ = 21.3° and 24°, and a relatively low intensity diffraction peak at 2θ = 19°.

Compared to the XRD curve of BW, it is found that the XRD curve of PEW (Figure 8, Curve II) also shows two high-intensity peaks at 2θ of 21.4° and 24°, which is similar to that of BW, but no additional peak at 2θ of 19° is observed. The two diffraction peaks at 2θ of 21.4° and 24° can be attributed to the (110) and (200) crystal planes of the orthorhombic crystal structure of PEW, respectively [56,57,58].

Compared to PEW, the XRD curves of PEW-*g*-MAH and PEW-*g*-MAH-MMA (Figure 8, Curve III, and Curve IV) show obvious reflection at the same diffraction angle, but the diffraction peak intensity decreases slightly and no new diffraction peak appears. This is consistent with the calculated results of crystallinity. Compared with the crystallinity of PEW, the crystallinity of PEW-*g*-MAH and PEW-*g*-MAH-MMA decrease (from 54% to 47% and 42%). These results show that the grafted MAH or MAH-MMA is mainly amorphous, and the increase in the number of disorder features may lead to the decrease in the local ordering of PEW that reduces the crystallinity of PEW-*g*-MAH and PEW-*g*-MAH-MMA, in agreement with the results of DSC analysis. 

## 4. Conclusions

A modified polyethylene wax with high melting point, high stability, and strong polarity suitable for the hot waxed wood process was prepared by MAH/MMA double monomer grafting method. The study on the acid value of PEW-*g*-MAH-MMA under different grafting conditions shows that for the MAH/MMA ratio of 1/1, the addition of MMA can effectively improve the reaction activity of MAH, increase the extent of anhydride grafting, and significantly increase the acid value. In addition, with the increase in the total amount of the monomer, the amount of BPO, and the reaction temperature, the acid value of PEW-*g*-MAH-MMA first increased and then decreased. The optimal formulation parameters and process conditions of the PEW-*g*-MAH/MMA system were determined and were the total amount of the monomer of 8 wt% and 2 wt% BPO, and the reaction temperature was 150 °C for the MAH/MMA ratio was 1/1. For these parameters, the acid value of PEW-*g*-MAH-MMA reached 18 KOH mg/g, which was consistent with beeswax and in accordance with the requirements set by the national standard, simulating the main chemical properties of beeswax. TGA and DSC analyses showed that the thermal stability and melting temperature of PEW-*g*-MAH-MMA were significantly higher than those of beeswax. The thermal stability of polyethylene wax was improved by MAH/MMA co-monomer grafting, but the melting temperature decreased slightly. FTIR and ^1^H-NMR analyses confirmed that MAH and MMA were copolymerized and grafted onto the macromolecular chain of polyethylene wax. The introduction of cyclic anhydride groups and ester groups endowed polyethylene wax with polar groups. XRD analysis showed that the crystal structure of PEW-*g*-MAH-MMA was similar to that of beeswax, and the grafted MAH-MMA was mainly amorphous. The crystallinity of polyethylene wax decreased slightly with the increase in the number of disorder features. This work developed an effective method for the preparation of modified polyethylene wax that simulates the chemical properties, chemical structure, and crystallization properties of beeswax, and can be used instead of beeswax in the wood hot waxing process, reduce the wax cost of hot waxing process, and meet the industrial demand of batch production. Considering the complexity of hot waxing wood used in outdoor environment, giving more functionality to polyethylene wax in future research should be considered.

## Figures and Tables

**Table 1:** Formulation and grafting temperature of the modified polyethylene wax.

Samples Code	Formulation				Grafting Temperature (°C)
	PEW	MAH	MMA	BPO	
1	100	0	0	0	150
2	100	4	0	2	150
3	100	4	2	2	150
4	100	4	4	2	150
5	100	4	8	2	150
6	100	4	16	2	150
7	100	2	2	2	150
8	100	8	8	2	150
9	100	16	16	2	150
10	100	4	4	1	150
11	100	4	4	3	150
12	100	4	4	4	150
13	100	4	4	2	130
14	100	4	4	2	140
15	100	4	4	2	160

Note: the formulation in Table 1 is expressed by weight parts.

**Table 2:** Test results of acid value of BW and PEW-*g*-MAH-MMA with different grafting conditions.

Groups	Weight Ratio (MAH/MMA)	Total Monomer Content (%)	BPO Content (%)	Reaction Temperature (°C)	Acid Value (KOH mg/g)
Group 1	1/0	8	2	150	4
	1/0.5				9.6
	1/1				18
	1/2				13.6
	1/4				1.6
Group 2	1/1	4	2	150	2
		8			18
		16			29
		32			24
Group 3	1/1	8	1	150	6.4
			2		18
			3		16
			4		13.6
Group 4	1/1	8	2	130	4.8
				140	11.2
				150	18
				160	13.6
BW	-	-	-	-	18
Standard	-	-	-	-	16–23

## Data Availability

All the data is available within the manuscript.
